# Using an intersectional framework to explore cannabis and tobacco co-use patterns: Protocol for a descriptive study

**DOI:** 10.1016/j.mex.2025.103565

**Published:** 2025-08-15

**Authors:** Bethany Shorey Fennell, Cherell Cottrell-Daniels, Kory Heier, Kristen McQuerry

**Affiliations:** a Department of Family & Community Medicine, College of Medicine, University of Kentucky, 2195 Harrodsburg Road, Lexington, KY, USA 40502; b Markey Cancer Center, College of Medicine, University of Kentucky, 750 Rose Street, Lexington, KY, USA; c Health Choice Network, 9064 NW 13th Terrace, Miami, FL 33172; d Department of Biostatistics, College of Public Health, University of Kentucky, 111 Washington Avenue, Lexington, KY, USA 40536

**Keywords:** Tobacco use, Cannabis use, Substance use, Cannabis and tobacco co-use

## Abstract

Cannabis and tobacco co-use is an emerging public health concern linked to increased dependence and reduced cessation success for both substances. However, little is known about how co-use patterns vary by product type, motivation, and demographic characteristics. This protocol outlines a cross-sectional study designed to characterize the frequency, type, and context of cannabis and tobacco co-use among U.S. adult tobacco users using an intersectional framework. A national sample of adults who used tobacco in the past 30 days (*N* = 3777) were recruited via CloudResearch Prime Panels. Participants completed detailed measures of tobacco and cannabis use, co-use behaviors, motivations, expectancies, health behaviors, and sociodemographic factors. This study employs a stratified sampling strategy to ensure representation across diverse geographic regions, racial and ethnic populations, and socioeconomic strata, and integrates robust data validation and quality control protocols. Results will help inform the development of targeted cessation interventions that address co-use, particularly among disproportionately impacted populations.

• Captures nuanced patterns of tobacco use, cannabis use, and tobacco and cannabis co-use across multiple product types.

• Examines co-use behaviors in relation to sociodemographic and psychosocial factors.

• Informs the development of tailored public health messaging and cessation interventions.

## Graphical abstract

**Figure:**
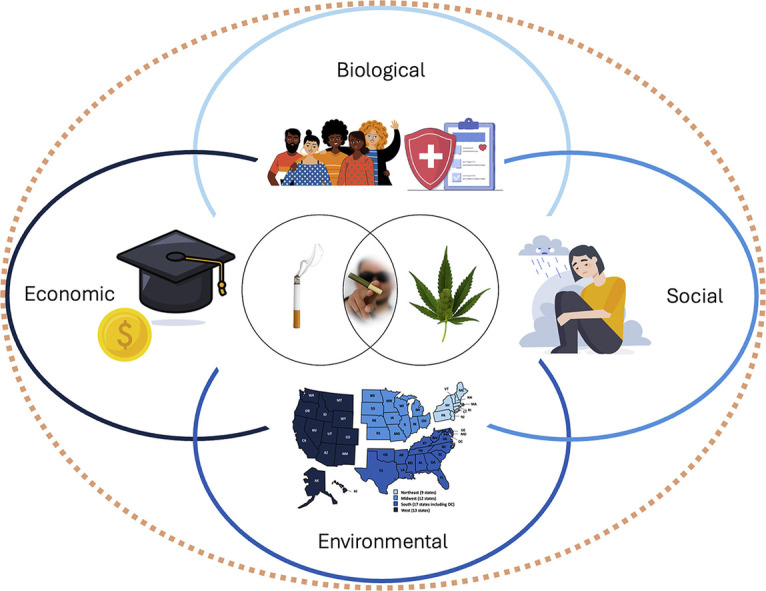

## Specifications table

**Table:** 

**Subject area**	Psychology
**More specific subject area**	Health behavior, substance use, risk assessment, decision making
**Name of your protocol**	Using an intersectional framework to explore cannabis and tobacco co-use patterns: Protocol for a descriptive study
**Reagents/tools**	Qualtrics software
**Experimental design**	Cross-sectional assessment of current cannabis and tobacco use, including questions relevant to public health, cancer prevention, and regulatory action.
**Trial registration**	Registered on Open Science Framework: Shorey Fennell, B., & Cottrell-Daniels, C. (2025, February 19). Tobacco and Cannabis Co-use. Retrieved from osf.io/a9tue *Currently Embargoed*
**Ethics**	All procedures were reviewed and approved by the University of Kentucky IRB. This research complied with ethical guidelines of the Declaration of Helsinki, including obtaining informed consent from all participants.
**Value of the Protocol**	• Describes frequency and quantity of tobacco and cannabis product use in a diverse sample of adults who use tobacco from all 50 US states and the District of Columbia. • Describes reasons for co-use and perceptions of different types of cannabis use • Describes associated health behaviors (alcohol use, exercise, other drug use), chronic health conditions, and sources of health information

## Background

An emerging public health concern is the increase of tobacco and cannabis co-use (co-use) among adults in the U.S. Among adults reporting tobacco use in the past month, the prevalence of also using cannabis increased from 18 % to 39 % between 2003 and 2012 [1]. Additionally, the prevalence of tobacco use among individuals who use cannabis increased from 69 % to 78 % between 2003–2012 [1]. Tobacco and cannabis co-use may increase dependence for both substances [2,3] and reduce the likelihood of successfully quitting either substance [4]. Further, there is evidence that co-use is associated with poorer outcomes in some subpopulations (e.g., young adults, Black/African American adults), with co-users experiencing more physical, mental, and social functioning harms than their peers who use tobacco only or cannabis only [5,6].

However, co-use is often assessed at a broad level (i.e., using both tobacco and cannabis in the past 30 days) and few research studies examined combustible and non-combustible forms of cannabis (e.g., blunts, edibles) and tobacco use (e.g., cigarettes, e-cigs/vapes) [5,7]. The primary aim of this investigation is to characterize co-use among U.S. adult tobacco users in a more nuanced way.

Evidence suggests that cannabis use varies by geographic region [8], gender [9], race/ethnicity [10,11], perceived riskiness [9], and the presence of health conditions such as chronic pain [12]. However, to our knowledge, no investigations have examined how co-use patterns, motivations, and perceptions may vary with multiple intersecting identities (e.g., co-use patterns of Black/African American adults in the Southern vs. Western U.S. regions).

**Fig. 1:**
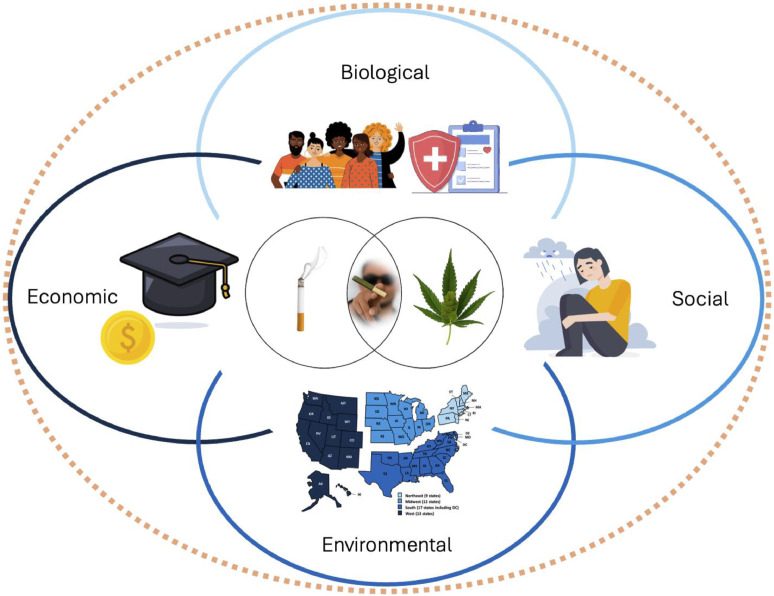
Considering intersectional relationships of tobacco and cannabis use and co-use among individuals with diverse biological, social, environmental, and economic characteristics and conditions. Image credits: Freepik.

This study was designed using an intersectional framework with a main goal of identifying patterns in tobacco and cannabis use and co-use that are embedded in participants’ interwoven characteristics and contexts (Fig. 1). Intersectionality Theory was initially developed by Kimberlé Crenshaw to describe how overlapping social identities may interact with structural and cultural systems of power (e.g., racism, the justice system) to produce unique inequities for some groups (i.e., Black women) [13,14]. More recently, Intersectionality Theory has been integrated into public health and substance use work [[15], [16], [17], [18], [19], [20], [21]]. The present study incorporates recommendations from Simkus and colleagues[16] to assess four domains associated with health outcomes for intersectional health equity research. These are 1) biological (e.g., age, race/ethnicity, sex), 2) social (e.g., stress, motives for use), 3) environmental (e.g., geography), and 4) economic (e.g., education, employment status).

This study responds to calls for a better understanding of cannabis and tobacco co-use and aims to:

1) Characterize patterns of co-use, including different types of cannabis and tobacco administration among U.S. adults who use tobacco.
2) Examine factors which may be associated with patterns of co-use behavior, such as geographic region, gender, race/ethnicity, risk perceptions, and other health behaviors.
3) Understand reasons for co-use and motivations and expectancies regarding cannabis use among adults who use tobacco.

Findings could enhance our understanding of different patterns of co-use among tobacco users[22] and inform the development of more effective tobacco cessation or cannabis cessation treatments [7,23].

## Description of protocol

**Experimental design**: Cross-sectional survey.

**Eligibility criteria:** 1) Adults aged 18 and older, 2) residence in the United States [U.S.], including the 50 states, Washington D.C., or Puerto Rico 3) are able to read and understand English 4) have used any form of tobacco/nicotine in the past 30 days. Participants were ineligible and directed out of the survey if they indicated they were under the age of 18, did not provide an age, did not reside in the U.S., did not choose a valid U.S. state or territory option, or reported not using any tobacco in the past 30 days. Table 1 describes recruitment targets specified to CloudResearch and actual recruitment.

**Table 1:** Recruitment targets specified to CloudResearch and actual participant recruitment.

Recruitment Sample Characteristics	Recruitment Targets Specified to Cloud Research	Actual Recruitment
% female	>45 %	49.2 %
% male	>45 %	50.2 %
% Non-Hispanic Black	∼20 %	22.0 %
% Hispanic of any race	∼20 %	8.0 %
% Non-Hispanic White	>40 %	58.7 %
U.S. Census Regions	“Good distribution” across U.S. Census Regions, similar to share of national population (Census Bureau, 2023)	
Northeast	Northeast – 17.0 %	Northeast – 16.7 %
South	South – 38.9 %	South – 45.0 %
Midwest	Midwest – 20.5 %	Midwest – 22.5 %
West	West – 23.6 %	West – 15.9 %

**Human subjects guidelines:** All research protocols and procedures were reviewed and approved by the University of Kentucky Institutional Review Board and followed the guidelines of the Declaration of Helsinki. Informed consent was obtained from interested individuals prior to entry into the survey.

**Recruitment:** Participants were recruited via CloudResearch Prime Panels, an online panel aggregator [24]. The study was advertised as a study on “Multiple Health Behaviors” rather than a tobacco-specific study to minimize selection bias and fraudulent entry to the study. Interested individuals were directed to the survey, hosted on Qualtrics, where they gave informed consent and then entered the main survey. The first part of the survey assessed inclusion criteria (age, U.S. residence, past month tobacco use). Ineligible participants were redirected out of the survey. Participants who met all inclusion criteria filled out detailed measures of tobacco/nicotine use, nicotine dependence, motivation and intentions to quit tobacco/nicotine, alcohol use, exercise, pain, cannabis use, other drug use, and chronic health conditions. Those who indicated past year cannabis use filled out additional detailed measures of cannabis use, cannabis-related problems, cannabis and tobacco co-use, cannabis use stereotypes, cannabis expectancies, and sources of cannabis information. At the end of the survey, participants were redirected via CloudResearch Prime Panels to the panel from which they entered the study and received the compensation from their original panel they agreed upon when entering the study. This varied, with some panels compensating participants with rewards points, money, or donations to a charity of their choice.

**Recruitment targets and actual recruitment:**

### Measures

The following measures were assessed during the survey. Scale and item scoring are included for reference. Any items designed to be reversed scored are designated with (r). Item names are shown in brackets.

Section 1 **Background Information**

*Instructions: First, we would like to get a little background information about you.*

**[age]** What is your age? __________

**[state]** In what state do you live? (dropdown box included all 50 U.S. States, Puerto Rico, and the District of Columbia)

*U.S. Census Region and Divisions were derived:*

**[NE]** Northeast Region

*New England Division:* Connecticut, Maine, Massachusetts, New Hampshire, Rhode Island, and Vermont
*Middle Atlantic Division:* New Jersey, New York, and Pennsylvania

**[MW]** Midwest Region

*East North Central Division:* Illinois, Indiana, Michigan,
Ohio, and Wisconsin
*West North Central Division:* Iowa, Kansas, Minnesota, Missouri,
Nebraska, North Dakota, and South Dakota

**[S]** South Region

*South Atlantic Division:* Delaware, District of Columbia, Florida,
Georgia, Maryland, North Carolina, South Carolina,
Virginia, and West Virginia
*East South Central Division:* Alabama, Kentucky, Mississippi and Tennessee
West South Central Division: Arkansas, Louisiana, Oklahoma and Texas

**[W]** West Region

*Mountain Division:* Arizona, Colorado, Idaho, Montana, Nevada,
New Mexico, Utah, and Wyoming
*Pacific Division:* Alaska, California, Hawaii, Oregon and Washington
Puerto Rico

**[zip]** What is your zip code? __________

The Index of Relative Rurality **[IRR]** (Waldorf & Kim, 2015)[25] was imputed from the 5-digit zip code.

**[race_ethn]** Which item(s) best describes your race/ethnicity? Please check all that apply.

0. White
1. Black or African American
2. Asian
3. Native Hawaiian or Other Pacific Islander
4. American Indian or Alaska Native
5. Hispanic/Latinx
98 Other (please specify): _______

**[gender]** How would you describe your gender identity?

0. Male
1. Female
2. Transgender female
3. Transgender male
4. Genderqueer / Genderfluid
98 Other (please specify): _________________

**[orient]** What is your sexual orientation?

0. Straight/Heterosexual
1. Gay or Lesbian/Homosexual
2. Bisexual
3. Prefer to self-describe ____________
98 Prefer not to say

**[educ]** What is the highest degree or level of school you have completed? If currently enrolled, highest degree received.

1. Grade school or less
2. Some high school, no diploma
3. High school diploma
4. GED or equivalent
5. Some college credit, no degree
6. Trade/technical/vocational training
7. Associate degree
8. College graduate (Bachelor’s degree)
9. Some graduate education
10. Graduate degree (MA, MS, MD, JD, PhD, etc.)

**[inc]** What is your total family income per year before taxes?

0. 0. Less than $10,000 per year or less than about $833 per month
1. $10,000 to $19,999 per year or less than about $1250 per month
2. $20,000 to $29,999 per year or less than about $2083 per month
3. $30,000 to $39,999 per year or less than about $2916 per month
4. $40,000 to $49,999 per year or less than about $3750 per month
5. $50,000 to $59,999 per year or less than about $4583 per month
6. $60,000 to $69,999 per year or less than about $5416 per month
7. $70,000 to $79,999 per year or less than about $6250 per month
8. $80,000 to $89,999 per year or less than about $7083 per month
9. $90,000 to $99,999 per year or less than about $7916 per month
10. $100,000 or more per year or more than $8333 per month
98 Refuse to Answer

**[employ]** What is your current employment status?

1. Full-time (work 30 or more hours per week)
2. Part-time (work less than 30 hours per week)
3. Unemployed
4. Disabled
5. Retired
6. Full-time student
7. Homemaker/stay-at-home parent
8. Other

**[marital]** What is your current marital status?

1. single
2. cohabitating
3. married
4. divorced
5. widowed

**[hshold]** With whom do you currently live? Please select all that apply

1. I live alone
2. Parent(s)
3. Spouse
4. Significant other or romantic partner
5. Your and/or your partner’s children
6. Roommate (non-romantic)

**[lang]** What language(s) do you speak at home? (select all)

1. English
2. Spanish
3. Other ______

**[literacy]** How confident are you filling out medical forms by yourself?

1. Not at all
2. Not very
3. Somewhat
4. Fairly
5. Very

Perceived stress

#### Perceived stress scale (PSS)

(Cohen et al., 1983) [26]

Scoring: Items should be averaged into a stress score **[pss]**, items to be reverse scored are indicated with (r). Higher scores represent more stress.

*Instructions: The following questions in this scale ask you about your feelings and thoughts during the last week. For each item, please choose the response that corresponds to how often you felt or thought that certain way.*

**[pss1]** In the last week, how often have you felt that you were unable to control the important things in your life?

1. Never
2. Almost never
3. Sometimes
4. Fairly often
5. Very often

**[pss2]** In the last week, how often have you felt confident about your ability to handle your personal problems? (r)

1. Never
2. Almost never
3. Sometimes
4. Fairly often
5. Very often

**[pss3]** In the last week, how often have you felt that things were going your way? (r)

1. Never
2. Almost never
3. Sometimes
4. Fairly often
5. Very often

**[pss4]** In the last week, how often have you felt difficulties were piling up so high that you could not overcome them?

1. Never
2. Almost never
3. Sometimes
4. Fairly often
5. Very often

Section 2 **Tobacco**

**[tob_prod]** Do you currently use any of the following tobacco/nicotine products? (check all that apply)

□ Cigarettes (prerolled or made with papers)
□ Cigars
□ e-cig/vape pen
□ Little cigars/cigarillos, bidis or black and milds
□ Pipe with tobacco
□ Dip/chew/snus
□ Hookah
□ Other ___
□ I don’t use tobacco

**[tob_rank]** Please rank the methods you use to take tobacco/nicotine from highest frequency at the top to lowest at the bottom. *Note: This item carried forward all indicated responses from tob_prod and had a drag and drop format to indicate frequency.*

□ Cigarettes (prerolled or made with papers)
□ Cigars
□ e-cig/vape pen
□ Little cigars/cigarillos, bidis or black and milds
□ Pipe with tobacco
□ Dip/chew/snus
□ Hookah
□ Other ___
□ I don’t use tobacco

**[tob_frq]** How often do you use [*each product selected in tob_prod*]? *Note: This item was asked for all indicated responses from tob_prod and had a corresponding item label (*e.g.*,* ***[tob_frq_cig]****).*

1. Daily
2. Weekly
3. Monthly
4. Yearly
5. In the past, but quit
6. Tried it once, but never again

**[tob_age]** At what age did you begin using [*each product selected above*]? ______________ *Note: This item was asked for all indicated responses from tob_prod and had a corresponding item label (*e.g.*,* ***[tob_age_cig]****).*

**[flavor]** In the past 30 days, what flavor(s) of [*each product selected above*] have you used? Choose all that apply. *Note: This item was asked for all indicated responses from tob_prod and had a corresponding item label (*e.g.*,* ***[flavor_cig]****).*

Tobacco-flavored
Menthol
Some other flavor (please specify)

**[cpd_ds]** How many cigarettes a day do you smoke on average? *Note: Those indicating daily cigarette smoking answered this question.*

**[cpd_nds]** On days you smoke cigarettes, how many cigarettes a day do you smoke on average? *Note: Those indicating nondaily cigarette smoking answered this question.*

**[smk_wake]** How soon after waking do you smoke your first cigarette? *Note: Those indicating smoking cigarettes answered this question.*

3. Within 5 minutes
2. 5–30 minutes
1. 31–60 minutes
0. 60+ minutes

**[cig100]** Have you smoked cigarettes 100 or more times in your life? *Note: Those indicating smoking cigarettes answered this question.*

1. Yes
2. No

**[smk_quit]** Are you planning to quit smoking? *Note: Those indicating smoking cigarettes answered this question.*

1. Within the next month
2. Within the next 6 months
3. Sometime in the future beyond 6 months
4. Not planning to quit
98. I do not smoke

**[tob_quit]** Are you planning to quit all tobacco use?

1. Within the next month
2. Within the next 6 months
3. Sometime in the future beyond 6 months
4. Not planning to quit

**[quitsmk_des]** Please rate your overall desire to quit smoking sometime in the future. *Note: Those indicating smoking cigarettes answered this question.*

1. No desire at all
2. Slight desire
3. Some desire
4. Moderate desire
5. Strong desire
98. I do not smoke

**[quittob_des]** Please rate your overall desire to quit all tobacco use sometime in the future.

1. No desire at all
2. Slight desire
3. Some desire
4. Moderate desire
5. Strong desire

**[smk_qt_att]** Have you ever tried to quit tobacco completely?

1. Yes
2. No

Contemplation

#### Contemplation ladder

(Biener & Abrams, 1991) [27]

**[ladder]** Below is a ladder with numbered steps/rungs ranging from 0 to 10. Each step/rung represents a thought about quitting tobacco/smoking. If you have smoked in the last month, please select a number from 0 to 10, 0 being 'Not thinking of quitting' to 10 'Taking action to quit'.

10: 10 Taking Action to quit (e.g. cutting down, enrolling in a program)
9: 9
8: 8 Starting to think about how to change my smoking patterns
7: 7
6: 6
5: 5 Think I should quit but not quite ready
4: 4
3: 3
2: 2 Think I need to consider quitting someday
1: 1
0: 0 No thought of quitting

**[nrt]** Are you currently using any tobacco/smoking cessation medications, including nicotine patch, nicotine gum, nasal spray, inhaler, lozenges, bupropion (Zyban), or varenicline (Chantix)?

1. No
2. Yes

Section 3 **Cannabis**

**[mj_ever]** Have you ever used cannabis/marijuana?

1. No
2. Yes

*Cannabis Products* - Adapted from Magnan & Ladd, 2019 and Cuttler & Spradlin, 2017 [28,29]

**[mj_prod]** Which methods do you use to take cannabis/marijuana? Please select all that apply.

0 = I do not use cannabis/marijuana
1 = Joints (using rolling papers)
2 = Blunts (cigar or little cigar sized joints with or without tobacco)
3 = Hand pipe
4 = Bong (water pipe)
5 = Hookah
6 = Vaporizer (e.g., Volcano, Vape pen)
7 = Edibles
8 = Concentrates (e.g., Oil, Wax, Shatter, Butane Hash Oil, Dabs, Tinctures)
9 = Other _______________________

**[mj_frq]** Which of the following best captures the average frequency you use cannabis currently?

0 = I do not use cannabis
1 = less than once a year
2 = once a year
3 = once every 3–6 months (2–4 times/yr))
4 = once every 2 months (6 times/yr)
5 = once a month (12 times/yr)
6 = 2 – 3 times a month
7 = once a week
8 = twice a week
9 = 3 – 4 times a week
10 = 5 – 6 times a week
11 = once a day
12 = more than once a day

Note: Those responding “I do not use cannabis” to both mj_prod and mj_frq were skipped to the bottom of the block and *did not receive futher cannabis questions.*

**[mj_rank]** Please rank the methods you use to take cannabis (marijuana) from highest frequency at the top to lowest at the bottom. *Note: This item carried forward all indicated responses from mj_prod and had a drag and drop format to indicate frequency.*

#### Cannabis flavors

Adapted from Watkins et al., 2023 [30] & Rose et al., 2020 [31]

*Note: Flavor items was asked for those indicating using hookah, vaporizer, concentrates, or blunts in mj_prod. Participants could select multiple responses. We did not assess flavor for edibles because nearly all commercially available edibles are flavored.*

**[hookmj_flav]** When you used hookah to use cannabis, what flavor do you usually use? *(only for those who indicate hookah use)*

**[vapemj_flav]** When you used a vaporizer to vape cannabis, what flavor did you usually use? *(only for those who indicate vaporizer use)*

**[concmj_flav]** When you used concentrates to use cannabis, what flavor did you usually use? *(only for those who indicate concentrates use)*

**[bluntmj_flav]** What flavor is your regular brand of cigar, cigarillo, or blunt wraps you smoke as a blunt with cannabis inside? *(only for those who indicate blunt use)*

1. Tobacco-flavored
2. Menthol
3. Mint
4. Clove or spice
5. Fruit
6. Chocolate
7. An alcoholic drink (such as wine, cognac, margarita, or other cocktails)
8. A non-alcoholic drink (such as coffee, soda, energy drinks, or other beverages)
9. Candy, desserts, or other sweets
10. Some other flavor (please specify)________________________________
11. Don't know

**[mj_days]** Approximately how many days of the past month did you use cannabis? Provide a number between 0–30. ____________

**[mj_social]** When you use cannabis/marijuana are you usually:

1. Alone
2. With friends/family
3. At a party
4. Other:___________

**[mj_wake]** How many hours after waking up do you typically first use cannabis?

7. within ½ hour of waking up
6. within 1 hour of waking up
5. 1 – 3 hours after waking up
4. 3 – 6 hours after waking up
3. 6 – 9 hours after waking up
2. 9 – 12 hours after waking up
1. 12 – 18 hours after waking up

**[mj_reason]** People sometimes use cannabis for medical/therapeutic reasons (e.g., pain management) and sometimes for recreational reasons (e.g., to get high, have fun). How would you describe your reasons for cannabis use?

1. Recreational
2. Medical
3. Both

**[mmj_pt]** Have you ever been registered as a Medical Marijuana Patient?

1. Yes
2. No
98. Prefer not to say

**[mj_qt_att]** Have you ever tried to quit cannabis completely?

1. Yes
2. No

**[mj_quit]** Are you planning to quit all cannabis use?

1. Within the next month
2. Within the next 6 months
3. Sometime in the future beyond 6 months
4. Not planning to quit

**[quitmj_des]** Please rate your overall desire to quit all cannabis use sometime in the future.

1. No desire at all
2. Slight desire
3. Some desire
4. Moderate desire
5. Strong desire

Section 4 **Other Health Behaviors**

**[aero_time]** During a typical week, how many minutes of aerobic exercise do you do? Aerobic exercise is any activity that uses large muscle groups, is done for at least 10 minutes each time, and is done at a level that causes your breathing to be heavy and your heart to beat faster (examples are running, swimming, bicycling, spinning, Zumba aerobics, basketball). _______________________

**[aero_type]** In the **PAST THREE MONTHS**, what types of aerobic activity have you engaged in? (circle all that apply)

1. Running
2. Biking/spinning
3. Zumba/HIIT/other aerobics
4. Sports (e.g., Basketball, Soccer, Tennis)
5. Swimming
6. Other (please list) ___________

**[str_time]** During a typical week, how many minutes of strength training do you do? Strength training, or resistance training, is a type of exercise that causes your muscles to work against outside resistance (examples are weightlifting and body weight resistance exercises, such as yoga). _______________________

**[str_type]** In the **PAST THREE MONTHS**, what types of strength training have you engaged in? (circle all that apply)

1. Weight lifting
2. Yoga/Pilates
3. CrossFit
4. Resistance bands
5. Other (please list) ___________

**[alc01]** Have you ever had a drink of alcohol?

1. No
2. Yes

**[alc3mo_frq]** In the *last 3 months*, how often did you consume *at least one* alcoholic drink**?**

1. Never
2. Occasionally
3. Once a month
4. 2–3 times a month
5. 4–5 times a month
6. Once a week
7. 2–3 times a week
8. 4–6 times a week
9. Every day

**[alc_wk]** During a typical week, how many alcohol drinks do you have? One “drink” is defined as one 12-ounce can or bottle of beer, one 5-ounce glass of wine, or one 1.5-ounce shot of hard liquor or spirits either by itself or in a mixed drink. _________________



http://www.niaaa.nih.gov/alcohol-health/overview-alcohol-consumption/what-standard-drink

**[alc3mo_quant]** In the *last 3 months*, how many drinks did you usually have at one time?

1. None
2. 1 drink
3. 2–3 drinks
4. 4–6 drinks
5. 7–9 drinks
6. 10–12 drinks
7. 13–15 drinks
8. 16–18 drinks
9. 19–20 drinks
10. More than 20 drinks

**[drunk]** In the *last 3 months*, when you drank alcohol how often did you get drunk?

1. Never
2. Almost never
3. Sometimes
4. Almost always
5. Always

**[subst_yr]** In the past year, which of the following substances have you used? Please select all that apply.

1. Prescription stimulants (Ritalin, Concerta, Dexedrine, Adderall, diet pills, etc.)
2. Inhalants (nitrous oxide, glue, gas, paint thinner, etc.)
3. Cocaine (coke, crack, etc.)
4. Methamphetamines (speed, crystal meth, ice, etc.)
5. Sedatives or sleeping pills (Valium, Serepax, Ativan, Xanax, Librium, Rohypnol, GHB, etc.)
6. Hallucinogens (LSD, acid, mushrooms, PCP, Special K, ecstasy, etc.)
7. Street opioids (heroin, opium, etc.)
8. Prescription opioids (fentanyl, oxycodone [Oxycontin, Percocet], hydrocodone [Vicodin], methadone, buprenorphine, etc.)
9. None of the above

**[subst_30]**

In the past 30 days which substances have you used? Please select all that apply. *Note: This item carried forward all indicated responses from subst_yr.*

1. Selected
0. Not selected
98. Prefer not to answer

**[tx]** Are you currently being treated for an alcohol or other drug problem?

0. No
1. Yes

Chronic pain: *Note: Chronic pain was defined as a pain problem lasting longer than three months and/or experiencing moderate or higher intensity pain (NRS ≥ 4) at least weekly for the past three months (Merskey & Bogduk, 1994)*[*32*]

**[pain]** Do you currently have a recurrent pain problem that has been present for 3 months or greater? Examples may include fibromyalgia, headaches, arthritis, Raynaud’s phenomenon, jaw, facial, shoulder, neck, leg, foot, arm, hand, chest, spine, back, or abdominal pain.

0. No
1. Yes

**[painfrq]** How frequently do you experience bodily aches and pains?

0. Not at all
1. Less than 1 time per month
2. 1–3 times per month
3. 1 time per week
4. 2–3 times per week
5. 3–6 times per week
6. Daily

**[painstr]** In general, how strong is your current pain?

0 = No pain
1
2
3
4
5
6
7
8
9
10= Worst pain possible

**[chronic]** Have you been diagnosed with any of the following? Please select all that apply. (yes/no)

Asthma
Anxiety
Cancer
Cardiac disease
Chronic pain
Depression
Diabetes
Dyslipidemia (high cholesterol)
HIV
Hypertension (high blood pressure)
Kidney disease
Lung disease
Post-Traumatic Stress Disorder
Stroke
Ulcers
None of the above
Other__________

*(for those who indicate cancer only)*

**[cancer]** What kind of cancer were you told that you had? Choose all that apply. (yes/no)

Bladder
Blood
Bone
Brain
Breast
Cervix (cervical)
Colon
Esophagus (esophageal)
Gallbladder
Kidney
Larynx/Windpipe
Leukemia
Liver
Lung
Lymphoma/Hodgkin's disease
Mouth/tongue/lip
Nervous system
Ovary (Ovarian)
Pancreas (pancreatic)
Prostate
Rectum (rectal)
Skin (melanoma)
Skin (non-melanoma)
Skin (don't know what kind)
Soft tissue (muscle or fat)
Stomach
Testis (testicular)
Throat
Thyroid
Uterus (uterine)
Other (please specify) ________________________

Note: *For those NOT eligible to participate in the additional questions for co-use*

**[honest_nocouse]**

Overall, how honest would you say you were in answering this questionnaire?

1= Not at all honest
2 = Not very honest
3 = Fairly honest
4 = Very honest
5 = Completely honest

Thank you for participating in our survey! We appreciate your time.

Note: *For those eligible to participate in the additional questions for co-use*

Now we are interested in hearing more about the perspectives of people who use tobacco and cannabis regularly. You've been invited to continue the survey because you indicated using cannabis and tobacco in the last year.

Section 5 **Detailed Cannabis-related Measures and Co-use Motives**

#### Cannabis administration stereotype measure

Adapted from McCool et al., 2004[33] & Butler et al., 2020[34]

Sum scores should be calculated for each substance (e.g., the count of “yes” responses for casm_joint, with responses indicated by (r) reverse scored before summation.)

Instructions: The following questions will ask what you think about people who use different types of cannabis. We are interested in the first thing that goes through your mind. This will be repeated for several types of cannabis. We appreciate your honest answer for each kind.

**[casm_joint01]** Take a moment and think about the type of person your age who uses joints (using rolling papers). In general, do you think that joint users your age tend to be…

**[casm_blunt01]** Take a moment and think about the type of person your age who uses blunts (cigar or little cigar sized joints with or without tobacco). In general, do you think that blunt users your age tend to be…

**[casm_pipe]** Take a moment and think about the type of person your age who uses handpipes. In general, do you think that handpipe users your age tend to be…

**[casm_bong]** Take a moment and think about the type of person your age who uses a bong (water pipe). In general, do you think that bong users your age tend to be…

**[casm_hookah]** Take a moment and think about the type of person your age who uses cannabis in hookah. In general, do you think that hookah users your age tend to be…

**[casm_vape]** Take a moment and think about the type of person your age who uses cannabis in vaporizers (e.g., Volcano, Vape pen). In general, do you think that vaporizer users your age tend to be…

**[casm_edible]** Take a moment and think about the type of person your age who uses edibles. In general, do you think that edible users your age tend to be…

**[casm_conc]** Take a moment and think about the type of person your age who uses concentrates (e.g., Oil, Wax, Shatter, Butane Hash Oil, Dabs, Tinctures). In general, do you think that concentrate users your age tend to be…

**Table:** 

	No	Yes
Independent	0	1
Sexy	0	1
Unattractive (r)	0	1
Cool	0	1
Trashy (r)	0	1
Intelligent	0	1
Immature (r)	0	1
Inconsiderate (r)	0	1
Like me	0	1

**[cudit]**

#### The cannabis use disorder identification test - revised (CUDIT-R)

Adamson et al., 2010 [35]

Instructions: Choose the response that is most correct for your cannabis use over the past six months.

*This questionnaire is scored by adding each of the 8 items:*

*Scores of 8 or more indicate hazardous cannabis use, while scores of 12 or more indicate a possible cannabis use disorder for which further intervention may be required.*

*Question 1–7 are scored on a 0–4 scale*

*Question 8 is scored 0,2, or 4*

















#### Brief cannabis motives measure (BCAMM)

Bartel et al., 2023 [36]

*[Scored on a sliding scale, Never = 0, Always = 100, slider default set to 50 for our study. Each item represents one domain. There is no total motives score, according to Bartel* et al.*]*

Instructions: Listed below are 6 reasons people might be inclined to use cannabis. Please decide how frequently your own cannabis use is motivated by each of the reasons listed and click on the place on the scale that best represents this frequency.

(enhancement motive) **[CAM_en]**

In the past 30 days, **I’ve used cannabis because it enhances positive feelings** (e.g., because I like the feeling, or to get a high).

(social motive) **[CAM_soc]**

In the past 30 days, **I’ve used cannabis because it’s a good way to socialize with others** (e.g., because it makes social gatherings more enjoyable or to be sociable).

(coping with anxiety motive) **[CAM_anx]**

In the past 30 days, **I’ve used cannabis because it helps me cope when I’m feeling nervous, anxious, or tense** (e.g., to reduce my anxiety or to relax).

9coping with depression motive) **[CAM_dep]**

In the past 30 days, **I’ve used cannabis because it helps me cope when I’m feeling sad, down, or blue** (e.g., because it helps me when I’m feeling depressed or to stop me from dwelling on things).

(conformity motive) **[CAM_conf]**

In the past 30 days**, I’ve used cannabis because I didn’t want to feel left out** (e.g., to be liked or to fit in with a group I like).

(expansion motive) **[CAM_exp]**

In the past 30 days, **I’ve used cannabis because it expands my awareness** (e.g., allows me to be more creative and original or to understand things differently).



#### Brief marijuana effect expectancy questionnaire (MEEQ-B)

Torrealday et al., 2008[37], pain expectancies by Shorey Fennell & Cottrell-Daniels

*Scoring: Can be looked at independently or combined into a two scale scores (positive or negative expectancies.*

*Impute variable* ***meeq_pos*** *where higher scores represent more positive expectancies by averaging responses. ('meeq2′, 'meeq3′, 'meeq4′, 'meeq7′)*

*Impute variable* ***meeq_neg*** *where higher scores represent more negative expectancies by averaging responses. ('meeq1′, 'meeq5′, 'meeq6′)*

**[meeq1]** Marijuana makes it harder to think and do things (harder to concentrate or understand; slows you down when you move).

  1. Disagree strongly
  2. Disagree somewhat
  3. Uncertain
  4. Agree somewhat
  5. Agree Strongly

**[meeq2]** Marijuana helps a person relax and feel less tense (helps you unwind and feel calm).

1. Disagree strongly
2. Disagree somewhat
3. Uncertain
4. Agree somewhat
5. Agree Strongly

**[meeq3]** Marijuana helps people get along better with others and it can help you feel more sexual (talk more; feel more romantic).

1. Disagree strongly
2. Disagree somewhat
3. Uncertain
4. Agree somewhat
5. Agree Strongly

**[meeq4]** Marijuana makes a person feel more creative and perceive things differently (music sounds different; things seem more interesting).

1. Disagree strongly
2. Disagree somewhat
3. Uncertain
4. Agree somewhat
5. Agree Strongly

**[meeq5]** Marijuana generally has bad effects on a person (you become angry or careless; after feeling high you feel down).

1. Disagree strongly
2. Disagree somewhat
3. Uncertain
4. Agree somewhat
5. Agree Strongly

**[meeq6]** Marijuana has effects on a person’s body and gives a person cravings (get the munchies/hungry; have a dry mouth; hard to stop laughing).

1. Disagree strongly
2. Disagree somewhat
3. Uncertain
4. Agree somewhat
5. Agree Strongly

**[meeq7]** Marijuana alleviates medical symptoms (e.g., pain, nausea).

1. Disagree strongly
2. Disagree somewhat
3. Uncertain
4. Agree somewhat
5. Agree Strongly

#### Tobacco/cannabis co-use motives

Akbar et al., 2019[38]

*Note: For participants with any cigarette use*

“The sum of these 8 items created a final scale with a possible range of 8–40.”

**[couse1_cigs]** My cigarette use increases when I’m using marijuana or I’m high.

1. Never
2. Occasionally
3. About Half of the Time
4. Often
5. Always

**[couse2_cigs]** I smoke a cigarette right after using marijuana (as a chaser).

1. Never
2. Occasionally
3. About Half of the Time
4. Often
5. Always

**[couse3_cigs]** Tobacco use improves my high from marijuana.

1. Strongly Disagree
2. Moderately Disagree
3. Neutral
4. Moderately Agree
5. Strongly Agree

**[couse4_cigs]** Tobacco makes me feel more alert when I’m high.

1. Strongly Disagree
2. Moderately Disagree
3. Neutral
4. Moderately Agree
5. Strongly Agree

**[couse5_cigs]** My marijuana and tobacco use are completely separate. (r)

1. Strongly Disagree
2. Moderately Disagree
3. Neutral
4. Moderately Agree
5. Strongly Agree

**[couse6_cigs]** When I am using marijuana or am high, my craving for cigarettes are greater.

1. Strongly Disagree
2. Moderately Disagree
3. Neutral
4. Moderately Agree
5. Strongly Agree

**[couse7_cigs]** When I am using marijuana or am high, my pleasure (or satisfaction) from smoking cigarettes is increased.

1. Strongly Disagree
2. Moderately Disagree
3. Neutral
4. Moderately Agree
5. Strongly Agree

**[couse8_cigs]** When I am smoking cigarettes, my pleasure (or satisfaction) from using marijuana is increased.

1. Strongly Disagree
2. Moderately Disagree
3. Neutral
4. Moderately Agree
5. Strongly Agree

#### Tobacco/cannabis co-use

adapted from Akbar et al., 2019[38]

*Note: For participants with no cigarette use*

**[couse1_nocig]** My tobacco use increases when I’m using marijuana or I’m high.

1. Never
2. Occasionally
3. About Half of the Time
4. Often
5. Always

**[couse2_nocig]** I use tobacco right after using marijuana (as a chaser).

1. Never
2. Occasionally
3. About Half of the Time
4. Often
5. Always

**[couse3_nocig]** Tobacco use improves my high from marijuana.

1. Strongly Disagree
2. Moderately Disagree
3. Neutral
4. Moderately Agree
5. Strongly Agree

**[couse4_nocig]** Tobacco makes me feel more alert when I’m high.

1. Strongly Disagree
2. Moderately Disagree
3. Neutral
4. Moderately Agree
5. Strongly Agree

**[couse5_nocig]** My marijuana and tobacco use are completely separate. (r)

1. Strongly Disagree
2. Moderately Disagree
3. Neutral
4. Moderately Agree
5. Strongly Agree

**[couse6_nocig]** When I am using marijuana or am high, my craving for tobacco is greater.

1. Strongly Disagree
2. Moderately Disagree
3. Neutral
4. Moderately Agree
5. Strongly Agree

**[couse7_nocig]** When I am using marijuana or am high, my pleasure (or satisfaction) from using tobacco is increased.

1. Strongly Disagree
2. Moderately Disagree
3. Neutral
4. Moderately Agree
5. Strongly Agree

**[couse8_nocig]** When I am using tobacco, my pleasure (or satisfaction) from using marijuana is increased.

1. Strongly Disagree
2. Moderately Disagree
3. Neutral
4. Moderately Agree
5. Strongly Agree

**[rel_risk]** Relative health risk perceptions

Is how you use marijuana less harmful, as harmful, more harmful than smoking cigarettes

-2 Much less harmful
-1 Less harmful
0 About the same level of harm
1 More harmful
2 Much more harmful
9 I don’t know

#### Information sources

Kruger et al., 2020[39], information trust adapted from Kruger et al., 2020 based on Figueiras et al., 2021[40]

1. How much information about cannabis/marijuana have you obtained from the following sources?

**[mjinfo1]** TV-None (1) A little (2) Some (3) A lot (4)
**[mjinfo2]** Social Media-None (1) A little (2) Some (3) A lot (4)
**[mjinfo3]** Family and Friends- None (1) A little (2) Some (3) A lot (4)
**[mjinfo4]** Newspaper/Magazines - None (1) A little (2) Some (3) A lot (4)
**[mjinfo5]** Radio- None (1) A little (2) Some (3) A lot (4)
**[mjinfo6]** Doctor or primary care provider- None (1) A little (2) Some (3) A lot (4)
**[mjinfo7]** Browsing the internet (e.g., websites)- None (1) A little (2) Some (3) A lot (4)
**[mjinfo8]** Your own experience None (1) A little (2) Some (3) A lot (4)
**[mjinfo9]** Dispensary None (1) A little (2) Some (3) A lot (4)
**[mjinfo10]** Medical cannabis caregiver None (1) A little (2) Some (3) A lot (4)
**[mjinfo11]** Books or journal articles None (1) A little (2) Some (3) A lot (4)

2. How much do you trust the information from these sources?

**[mjtrust1]** TV- None (1) A little (2) Some (3) A lot (4)
**[mjtrust2]** Social Media- None (1) A little (2) Some (3) A lot (4)
**[mjtrust3]** Family and Friends- None (1) A little (2) Some (3) A lot (4)
**[mjtrust4]** Newspaper- None (1) A little (2) Some (3) A lot (4)
**[mjtrust5]** Radio- None (1) A little (2) Some (3) A lot (4)
**[mjtrust6]** Doctor or primary care provider- None (1) A little (2) Some (3) A lot (4)
**[mjtrust7]** Browsing the internet (e.g., websites)- None (1) A little (2) Some (3) A lot (4)
**[mjtrust8]** Your own experience None (1) A little (2) Some (3) A lot (4)
**[mjtrust9]** Dispensary None (1) A little (2) Some (3) A lot (4)
**[mjtrust10]** Medical cannabis caregiver None (1) A little (2) Some (3) A lot (4)
**[mjtrust11]** Books or journal articles None (1) A little (2) Some (3) A lot (4)

**[tob_subst]** When you have tried to quit using cannabis completely, did your use of tobacco change? *(Only for those who have ever tried to quit cannabis)*

1 Yes, it increased
0 No, it stayed the same
-1 Yes, it decreased

**[can_subst]** When you have tried to quit using tobacco completely, did your use of cannabis change? *(Only for those who have ever tried to quit tobacco)*

1 Yes, it increased
0 No, it stayed the same
-1 Yes, it decreased

**[honest_couse]** Overall, how honest would you say you were in answering this questionnaire?

1. Not at all honest
2. Not very honest
3. Fairly honest
4. Very honest
5. Completely honest

Thank you for participating in our survey! We appreciate your time.

*Note: Six attention checks were embedded throughout the survey, 4 in Sections 1–4 and 2 in Section 5. See “Automatic Exclusions” below for more detail.*

## Timeline

**Fig. 2:**
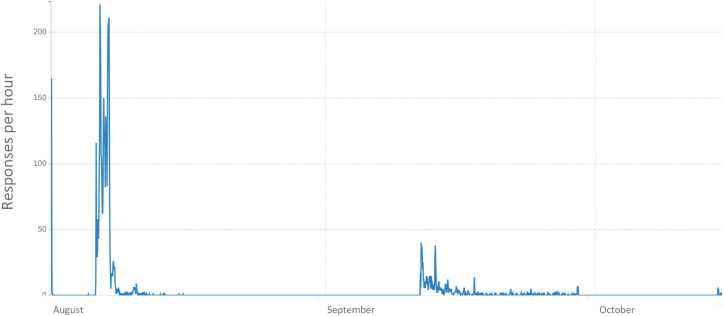
Survey responses per hour, August – October 2024.

Data collection began on 06 August 2024 and concluded 15 October 2024. Fig. 2 shows the timeline of accrual.

## Data quality

Based on the PI’s prior experience with online panel recruitment[41,42] rigorous data quality measures were employed during participant recruitment and data cleaning procedures.

First, CloudResearch has developed Sentry, a proprietary data quality and bot detection checker, which vets interested respondents before they enter the survey. Only high-quality participants are allowed to access the survey link.

Second, throughout recruitment, the PI (BSF) periodically monitored the data for quality, ensuring that eligibility criteria were met and that a sufficient number of attention check questions were answered appropriately. Any participants whose data did not meet the criteria under “Automatic Exclusions” below or who did not pass sufficient attention checks were replaced by CloudResearch through their data quality guarantee.

Finally, detailed quality check procedures and data cleaning were followed prior to analysing data. These included:

### Automatic exclusions

- Did not agree to participate. (variable “consent” = 2)
- Did **not** fill out or meet required demographic inclusion criteria which are:
  ○ Age is filled
  ○ Age (≥18)
  ○ State is filled
  ○ State is not “I do not reside in the U.S.”
- Did not report tobacco use.
  ○ Respondents were excluded if in variable “tob_prod” 1) they selected “I don’t use tobacco” (variable “tob_prod_9”) *and* did not choose any other product or 2) they did not select any product.

Note: Participants who did not agree to participate were directed out of the survey at the consent page. Participants who did not fill out required demographic information (age, U.S. State) or who provided ineligible answers (e.g., age < 18, State is “I do not reside in the U.S.”) were directed out of the survey after Section 1. Participants who did not report tobacco use were directed out of the survey at the beginning of Section 2.

- Did not pass Attention Checks.
  ○ Respondents who use tobacco only: Must pass 3 of 4 attention checks embedded in Sections 1–4 (checks 1–4). They did not move on to Section 5.
  ○ Respondents who co-use cannabis and tobacco: Must pass 4 of 6 attention checks, including at least one of the checks in Section 5 (checks 5 & 6).

These variable names and response values are:

**[check1]** – “Choose "Almost Never" for this response”.
  Correct = “Almost Never”, Value = 2, Failed = Values 1,3,4,5
**[check2]** – “Were you born on planet earth?”
  Correct = Yes, Value = 1, Failed = No, Value = 0
**[check3]** – Leave this response option blank.
  Correct = Blank open response field. Failed = filled open response field.
**[check4]** – “Answer "Sometimes" to this question”.
  Correct = “Sometimes”, Value = 2. Failed = Values 1,3
**[check5]** – For this question, choose "Weekly" as your response.
  Correct = “Weekly”, Value = 3. Failed = Values 0,1,2,4
**[check6]** - Choose "Agree Strongly" for this response.
  Correct = “Agree Strongly”, Value = 5. Failed = Values 1,2,3,4

- Quit early in the survey.
  ○ Respondents who quit prior to filling out the tobacco (Section 2) and cannabis use questions (the first two questions of Section 3) were excluded.
- Were not honest.
  ○ Participants who indicated they were not honest in [honest_nocouse] or [honest_couse] were excluded. These read: “Overall, how honest would you say you were in answering this questionnaire?” To be included, the response value had to be “Fairly honest”, “Very honest”, or “Completely honest”. Those responding “Not very honest” or “Not at all honest” were excluded.
- Were too speedy.
  ○ Respondents were excluded if time to complete the survey was less than 2 minutes (120 seconds).

### Potential exclusions

We also examined indicators that may, in conjunction with one another, point to fraudulent or bad quality data. Participants were excluded if they had more than one of these.

- Strange or repetitive answers to open-response questions, which can indicate bot activity
- Inappropriate responses (e.g., age = 2024 or 300).
- Straightlining (i.e., many answers of the same value in a row) which can indicate inattentiveness.

**Fig. 3:**
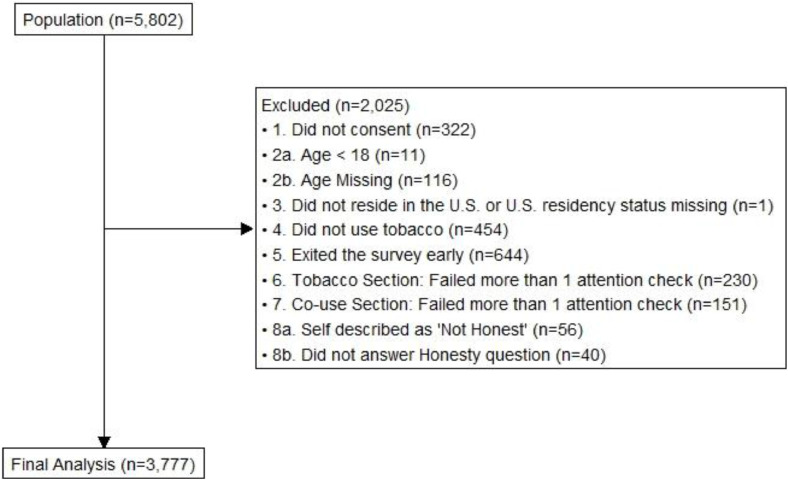
Survey consort flow chart.

Fig. 3 displays reasons for exclusion from the final sample.

## Protocol validation

Overall sample characteristics are displayed in Table 2.

**Table 2:** Sample Characteristics.

(*N* = 3777)	*M* (*SD*) or n ( %)
**Age**	
Mean (SD)	47.9 (16.4)
Median [Q1, Q3]	49.0 [34.0, 62.0]
18–29	653 (17.3 %)
30–54	1581 (41.9 %)
55+	1543 (40.9 %)
**Gender**	
Female	1852 (49.09 %)
Male	1897 (50.28 %)
Transgender/Genderqueer/Other	24 (0.64 %)
**Sexual Orientation**	
Straight/Heterosexual	3348 (88.64 %)
Lesbian or Gay	156 (4.13 %)
Bisexual	248 (6.57 %)
Prefer to self-describe	13 (0.34 %)
Prefer not to say	12 (0.32 %)
**Race/Ethnicity**	
American Indian or Alaskan Native	53 (1.40 %)
Asian	96 (2.54 %)
Black/African American	824 (21.82 %)
Hispanic/Latinx	294 (7.79 %)
Multiracial	255 (6.75 %)
Native Hawaiian or Other Pacific Islander	12 (0.32 %)
White	2223 (58.87 %)
Other	19 (0.50 %)
**U.S. Census Region**	
Northeast	629 (16.65 %)
South	1698 (44.96 %)
Midwest	849 (22.48 %)
West	601 (15.91 %)
**Education**	
Less than high school	282 (7.48 %)
High school diploma/GED	1270 (33.67 %)
Some college/vocational degree	1464 (38.81 %)
Bachelor’s degree or higher	756 (20.04 %)
**Health Literacy**	
Inadequate	745 (19.74 %)
Adequate	3029 (80.26 %)
**Income**	
Under $20,000	1049 (28.35 %)
$20,000 - 49,999	1297 (35.05 %)
$50,000 and above	1354 (36.59 %)
**Employment**	
Employed	1984 (52.53 %)
Unemployed	403 (10.67 %)
Retired/Unable to work	1094 (28.96 %)
Student/Homemaker/Other	296 (7.84 %)
**Marital Status**	
Single	1347 (35.74 %)
Cohabitating/Married	1596 (42.35 %)
Divorced/Widowed	826 (21.92 %)
**Live alone?**	
Live Alone	1082 (28.66 %)
Other	2693 (71.34 %)
**Live with kids?**	
Without children	3400 (90.07 %)
Your and/or your partner's children	375 (9.93 %)
**# of Chronic Conditions**	
Mean (SD)	2.0 (1.9)
Median [Q1, Q3]	2.0 [0.0, 3.0]
**Chronic Conditions (n, % yes)**	
Asthma	652 (17.26 %)
Cancer	186 (4.92 %)
Cardiac Disease	177 (4.69 %)
Chronic Pain	743 (19.67 %)
Diabetes	545 (14.43 %)
Dyslipidemia (high cholesterol)	248 (6.57 %)
HIV	49 (1.30 %)
Hypertension	832 (22.03 %)
Kidney Disease	102 (2.70 %)
Lung Disease	110 (2.91 %)
Stroke	145 (3.84 %)
Ulcers	139 (3.68 %)
Anxiety	1563 (41.38 %)
Depression	1327 (35.13 %)
PTSD	477 (12.63 %)
None of the above	990 (26.21 %)
Other	187 (4.95 %)
**# of Tobacco Products**	
1	2292 (60.68 %)
2	922 (24.41 %)
3	316 (8.37 %)
4	151 (4.00 %)
5	58 (1.54 %)
6	18 (0.48 %)
7	20 (0.53 %)
8	0 (0.00 %)
**Tobacco Products (n, % yes)**	
Cigarettes (prerolled or made with papers)	3090 (81.81 %)
Cigars	665 (17.61 %)
E-cig/vape pen	1158 (30.66 %)
Little cigars/cigarillos, bidis or black and milds	585 (15.49 %)
Pipe with tobacco	218 (5.77 %)
Dip/chew/snus	158 (4.18 %)
Hookah	285 (7.55 %)
Other	67 (1.77 %)
**Age initiated tobacco**	
Mean (SD)	19.3 (8.5)
Median [Q1, Q3]	17.0 [15.0, 21.0]
**Age initiated smoking (for cigarette users)**	
Mean (SD)	18.1 (6.6)
Median [Q1, Q3]	17.0 [15.0, 20.0]
**Ever tried to quit all tobacco**	
No	1067 (28.26 %)
Yes	2709 (71.74 %)
**Intent to Quit Tobacco**	
Within the next month	509 (13.48 %)
Within the next 6 months	877 (23.22 %)
Sometime in the future beyond 6 months	1258 (33.31 %)
Not planning to quit	1133 (30.00 %)
**Past 30-day Cannabis Use**	
No	1939 (51.34 %)
Yes	1838 (48.66 %)

Participants were recruited from all 50 U.S. States and Washington D.C. Table 3 displays the geographic distribution of the analytic sample.

**Table 3:** Geographic distribution of participants within the United States.

State or territory	
Alabama	72 (1.91 %)
Alaska	3 (0.08 %)
Arizona	65 (1.72 %)
Arkansas	40 (1.06 %)
California	252 (6.67 %)
Colorado	42 (1.11 %)
Connecticut	40 (1.06 %)
Delaware	13 (0.34 %)
District of Columbia	15 (0.40 %)
Florida	270 (7.15 %)
Georgia	145 (3.84 %)
Hawaii	10 (0.26 %)
Idaho	13 (0.34 %)
Illinois	140 (3.71 %)
Indiana	87 (2.30 %)
Iowa	31 (0.82 %)
Kansas	28 (0.74 %)
Kentucky	83 (2.20 %)
Louisiana	80 (2.12 %)
Maine	16 (0.42 %)
Maryland	59 (1.56 %)
Massachusetts	44 (1.16 %)
Michigan	139 (3.68 %)
Minnesota	40 (1.06 %)
Mississippi	50 (1.32 %)
Missouri	70 (1.85 %)
Montana	8 (0.21 %)
Nebraska	12 (0.32 %)
Nevada	63 (1.67 %)
New Hampshire	8 (0.21 %)
New Jersey	84 (2.22 %)
New Mexico	35 (0.93 %)
New York	234 (6.20 %)
North Carolina	145 (3.84 %)
North Dakota	10 (0.26 %)
Ohio	220 (5.82 %)
Oklahoma	73 (1.93 %)
Oregon	37 (0.98 %)
Pennsylvania	176 (4.66 %)
Puerto Rico	0 (0.00 %)
Rhode Island	7 (0.19 %)
South Carolina	69 (1.83 %)
South Dakota	4 (0.11 %)
Tennessee	93 (2.46 %)
Texas	365 (9.66 %)
Utah	15 (0.40 %)
Vermont	7 (0.19 %)
Virginia	83 (2.20 %)
Washington	55 (1.46 %)
West Virginia	56 (1.48 %)
Wisconsin	68 (1.80 %)
Wyoming	3 (0.08 %)

## Qualtrics survey flow













### Limitations

Because of the cross-sectional nature of the survey, we cannot make causal conclusions about the relationship between tobacco and cannabis use among adults who use tobacco. Rather, we attempt to capture current tobacco and cannabis use, perceptions, intentions and motivation to quit either substance, and current chronic health outcomes that may be associated with different patterns of current use. Additionally, although we captured a diverse sample in terms of age, gender, race/ethnicity, and geography throughout the U.S., our online recruitment approach precludes some individuals from participating (e.g., those without internet or mobile phone access). Additionally, we planned to recruit ∼20 % Hispanic/Latino participants and, despite a concerted effort in September and October (see Fig. 2) we were unable to meet this target. The survey and recruitment materials were only offered in English, which may have limited our ability to effectively reach this population.

## CRediT author statement

**Bethany Shorey Fennell**: Conceptualization, Methodology, Software, Validity tests, Data curation, Writing- Original draft preparation, Visualization, Investigation, Supervision

**Cherell Cottrell-Daniels:** Conceptualization, Methodology, Writing- Reviewing and editing

**Kory Heier:** Data curation, Analysis

**Kristen McQuerry:** Data curation, Analysis

## Related research article

None

## Declaration of competing interest

The authors declare that they have no known competing financial interests or personal relationships that could have appeared to influence the work reported in this paper.

## Data Availability

Data will be made available upon request after the embargo period specified in the OSF registration of the project.
